# The Efficiency of Multimodal Opioid-Free Anesthetic Management in a Patient Undergoing Major Abdominal Surgery - Case Report

**DOI:** 10.31480/2330-4871/206

**Published:** 2025-08-14

**Authors:** Manuel Alejandro Fierro, Cristina Chandler, Amy Chen, Hong Liu, Artin Yeranossian

**Affiliations:** Department of Anesthesiology and Pain medicine, University of California, Davis Health, Sacramento, USA

**Keywords:** Opioid free anesthesia, Local anesthetics, Laparoscopic surgery

## Abstract

Opioid-free anesthesia has become a purposeful alternative to addressing nociception in the perioperative settings. Increased opioid availability has been accompanied by an opioid crisis. Around 1.6 million Americans had an opioid dependence problem in 2019 with 70,000 people dying from overdose every year. The concept of multimodal anesthesia aims to avoid the negative effect of opioid use intraoperatively on the patient’s postoperative outcomes. Though adverse sequelae such as ileus, respiratory depression, somnolence, immunosuppression and hyperalgesia are well documented in the literature, the use of diverted prescription opioids can result in addiction or fatal overdose. In recent years concerned researchers and physicians tried to identify practical strategies to a more cautious opioid use and even an opioid free approach. A multidisciplinary perioperative care plan that includes a preoperative evaluation, an intraoperative and postoperative care strategy needs to be formulated. In this case report, we describe pertinent considerations in tailoring a successful opioid sparing analgesia technique that provided superior pain relief using multiple interventions of local anesthetics (MILANA) for a patient undergoing a complex abdominal surgery.

## Background

Perioperative opioids use is associated with respiratory depression, impaired gastrointestinal function, delirium and the potential development of opioid addiction [1]. The United States is facing an opioid crisis with an epidemic of prescription opioid abuse and deaths from overdose [2]. The Enhanced recovery after surgery (ERAS) protocols emphasize minimizing opioid use in perioperative care to enhance recovery, reduce complications, and accelerate functional recovery. Opioid-free anesthesia (OFA) is gaining prominence in gastrointestinal surgeries, with focus on maintaining preoperative organ function and reducing the significant stress response postoperatively [1]. We report the case of a 60-year-old woman with a history of Crohn’s disease, who underwent elective ileum and cecum resection for bowel obstruction due to strictures. The patient requested a non-opioid multimodal pain management, believing this would help her recover faster and expedite bowel movement return, especially given her unintentional weight-loss state. The successful multimodal pain management incorporated a combination of analgesic modalities, including medications, regional interventions and non-pharmacologic techniques. The discussion will review the evidence-based choice of multimodal analgesia regimen, the comparison in recovery timeline with opioid-based management of similar surgeries, andconcerns surrounding potential local anesthetic systemic toxicity (LAST).

## Case Presentation

### Preoperative assessment

The patient was a 60-year-old woman, weighing 60 kilograms (kg), with a long-standing history of Crohn’s disease, which had been managed medically with azathioprine and infliximab, until the development of bowel obstruction from strictures. She presented with symptoms of nausea, vomiting, abdominal distention, and weight loss. A computer tomography (CT) scan revealed severe ileal and cecal narrowing, and the decision was made to proceed with elective ileocecal resection. Patient denied any other significant past medical history or any allergies. Vital signs and blood tests were within normal limits, except for hypoalbuminemia. Physical exam depicted a malnourished patient, looking tired with pale and dry skin. Patient denied any depression or poor concentration. Preoperative counselling included a detailed discussion regarding the perioperative pain management. Patient denied having any complex pain problem. Moreover, the patient expressed a desire in receiving an opioid sparing analgesic approach, which canpromote early return of usual function (mobilization, eating and drinking).

### Intraoperative management

Patient was taken to the operating room, where she received the American Society of Anesthesiologists (ASA) standard monitoring including electrocardiogram (ECG) non-invasive blood pressure (NBP), oxygenation, respiration and temperature. The NBP was 100/54 mmHg, heart rate (HR) was 97 beats/min, and oxygen saturation (SO2) was 95%. After 3 minutes of effective preoxygenation, the induction was performed using Propofol 100 milligrams (mg), fentany l50 micrograms (mcg), lidocaine 40 mg, and rocuronium 60 mg. After intubation with 7.0 size endotracheal tube, a second 18 g intravenous (iv) catheter was placed in the right hand, followed by a right-hand radial artery catheterization for invasive blood pressure monitoring. Throughout the procedure, the patient received a balanced anesthesia, sevoflurane-based, using a non-opioid pain approach with lidocaine at a rate of 2 mg/kg/hour(hr) and dexmedetomidineat 0.2 mcg/kg/hr infusions, acetaminophen 1 gram (g) iv, and magnesium sulfate 2 g iv. Normothermia was maintained for the entire procedure. Surgical procedure lasted 6 hours. Patient received a total of 2,500 milliliters (ml) of crystalloids, and 500 ml of Albumin 5%. Blood loss was 350 ml. At the conclusion of surgery, before extubation, bilateral transversus abdominis plane (TAP) block catheters were then placed using saline bolus, and a continuous infusion of 0.2% ropivacaine was started at a rate of 10 mL per hour into each catheter to manage postoperative pain [3]. The careful timing of lidocaine discontinuation 1 hour prior to placement of TAP block ensured a smooth transition between systemic analgesia and regional anesthesia, minimizing the cumulative dose of local anesthetics and mitigating LAST risk [4]. The anesthesia team remained prepared to treat any signs of LAST with iv lipid emulsion therapy, though no complications occurred. Patient was successfully extubated and taken to recovery room.

### Postoperative course

Upon emergence from anesthesia, the patient initially reported a numerical rating scale (NRS) pain score of 7 out of 10. The TAP block infusions were increased from 10 mL/hr to 12 mL/hr, and within two hours, her NRS pain score improved to 1 out of 10. After discharging from post anesthesia care unit (PACU), patient was transferred to medical-surgical unit where she received around the clock 1 g oral acetaminophen every six hours for analgesia [5]. This perioperative anti-nociception opioid sparing approach, combining systemic and regional techniques, proved effective in managing the patient’s pain. Twenty-four hours after surgery, the patient’s pain fluctuated between 4 and 5 out of 10. By the second postoperative day (POD), patient reported a pain score of 2-3 out of 10, which allowed her to begin early mobilization and initiate a liquid diet. The patient’s bowel function returned within 48 hours, significantly earlier than the typical 3-5 days observed in patients receiving opioids [6]. Patient was discharged on POD4.

## Discussion

### Multimodal analgesia

The successful management of this patient’s pain without opioids underscores the importance of a multimodal analgesia approach in ERAS protocols. Several modalities used for this patient (lidocaine, dexmedetomidine, magnesium sulphate and acetaminophen) complemented each other, further reducing pain intensity and enhancing the efficiency of the regional block [1]. Lidocaine infusions are increasingly recognized as an effective adjunct in opioid-sparing strategies and provide analgesia through inhibition of neuronal pain transmission, reduction of inflammatory and neuropathic pain, and mitigate the need for high-dose local anesthetics in peripheral nerve blocks [7]. According to a large analysis of forty-five clinical trials, perioperative lidocaine infusion correlated with decreased visual analogue scale (VAS) pain scores at 1 to 4 hours and 24 hours postoperatively, decreased opioid requirements and hospital length of stay, reduced nausea, and shortened time to first flatus when compared to control patients. It concluded that patients undergoing laparoscopic and open abdominal surgery benefitted the most from perioperative iv lidocaine infusions [8-10]. LAST is a potential complication of regional anesthesia, particularly when large volumes of local anesthetics are administered or absorbed into the systemic circulation [11]. The coordinated management of the lidocaine infusion by the acute pain service ensured a seamless transition to the TAP block while minimizing the risk of LAST, which can occur when high doses of local anesthetics are administered simultaneously or in close succession [12,13].

Dexmedetomidine works as an alpha-2 agonist to achieve sympatholysis, sedation, and analgesia [14]. It is thought to be up to ten times more potent than clonidine. Dexmedetomidine is known to reduce opioid requirements by modulating pain pathways and attenuating sympathetic responses through alpha-2 receptor agonism.While a powerful hypnotic and sedative, dexmedetomidine typically does not negatively impact ventilation. Loading doses can, however, cause hypotension or bradycardia. Some studies suggest that an opioid-free general anesthesia based on dexmedetomidine could be effective; however, prolonged time to extubation and cardiovascular complications are associated with dexmedetomidine [15]. Magnesium acts as an NMDA receptor antagonist, enhancing analgesia and reducing opioid consumption [15]. Acetaminophen inhibits the production of prostaglandins in the brain, which are chemicals involved in pain and inflammation. By adding it to the mixture, pain is not only better controlled, but also reduces the risks associated with opioid usage [1].

### Early Return of bowel function and recovery time

The early return of bowel function is a key objective of the OFA regimen, particularly in major bowel surgery. In opioid-based analgesic regimens, the return of gastrointestinal function is often delayed due to the decrease in its motility caused by opioids [16]. In this case, the multimodal approach incorporating lidocaine, dexmedetomidine, magnesium, acetaminophen, and TAP blocks facilitated a faster return of bowel function and shortened the overall recovery period. The patient was discharged from the hospital on POD 4, which is shorter than the average length of stay of 5-7 days typically seen in opioid-based pain management after gastrointestinal surgeries [17,18]. In contrast to the OFA pain management, some research supports the opioid-sparing strategies which can improve outcomes in gastrointestinal surgeries, particularly in patients who are at risk for opioid-related complications [19]. A meta-analysis aimed at exploring the impact of OFA on pain dependent recovery time and opioid consumption in patients undergoing bariatric surgery found that OFA was associated with a lower incidence of nausea and vomiting, faster recovery due to lower pain score and less opiate consumption in the PACU [20].

Other studies did not support this claim.In the SOFA randomized, controlled clinical trial [21], the patients undergoing the OFA protocol for elective surgery had statistically significant, but not clinically meaningful, improved quality of recovery compared to patients in the standard group. Another randomized clinical trial with patients undergoing radical colectomy using pain threshold monitoring showed no difference in intraoperative pain threshold readings; however, non-opioid anesthesia did reduce the rescue analgesic consumption after surgery [22]. With respect to the quality of recovery a study, another randomized study that looked at the impact of OFA on postoperative quality of recovery in patients after laparoscopic cholecystectomy showed no significant differences in the duration of PACU stay, duration of extubation and the incidence of bradycardia [23].

While OFA may be attractive to some patients, adjuvant drugs have their own potential adverse effects, such as the hypotension and bradycardia with dexmedetomidine and risk of local anesthetic systemic toxicity with lidocaine infusions. Furthermore, use of these adjuvant infusions necessitates monitoring, require equipment and additional nursing training, and increase costs. Therefore, the risk-benefit analysis should be done for each patient when considering an opiate-free anesthetic.

## Conclusion

The choice of OFA multimodal approach to pain management emerges as a popular alternative, especially in patients undergoing major bowel surgery. The idea of using a multimodal perioperative analgesia protocol that combined nonopioid drugs, and regional techniques allowed the achievement of a good quality general anesthesia, while avoiding the unwanted postoperative outcomes commonly seen with opioids use. By incorporating drugs whose action complement each other: alpha-2 antagonists (dexmedetomidine) with NMDA antagonists (lidocaine, magnesium sulphate) and sodium channel blockers (local anesthetics), this patient had good postoperative outcomes related to pain, function and quality of life. However, all these drugs have known side-effects. And although the principle of OFA is gaining popularity among the anesthesia specialists, careful management of the drug combination with closely monitoring of potential adverse reactions (e.g. LAST), is highly recommended. This case suggests that, with appropriate patient selection and monitoring, OFA can be a safe and effective strategy in open colorectal surgery.

## Figures and Tables

**Figure 1:**
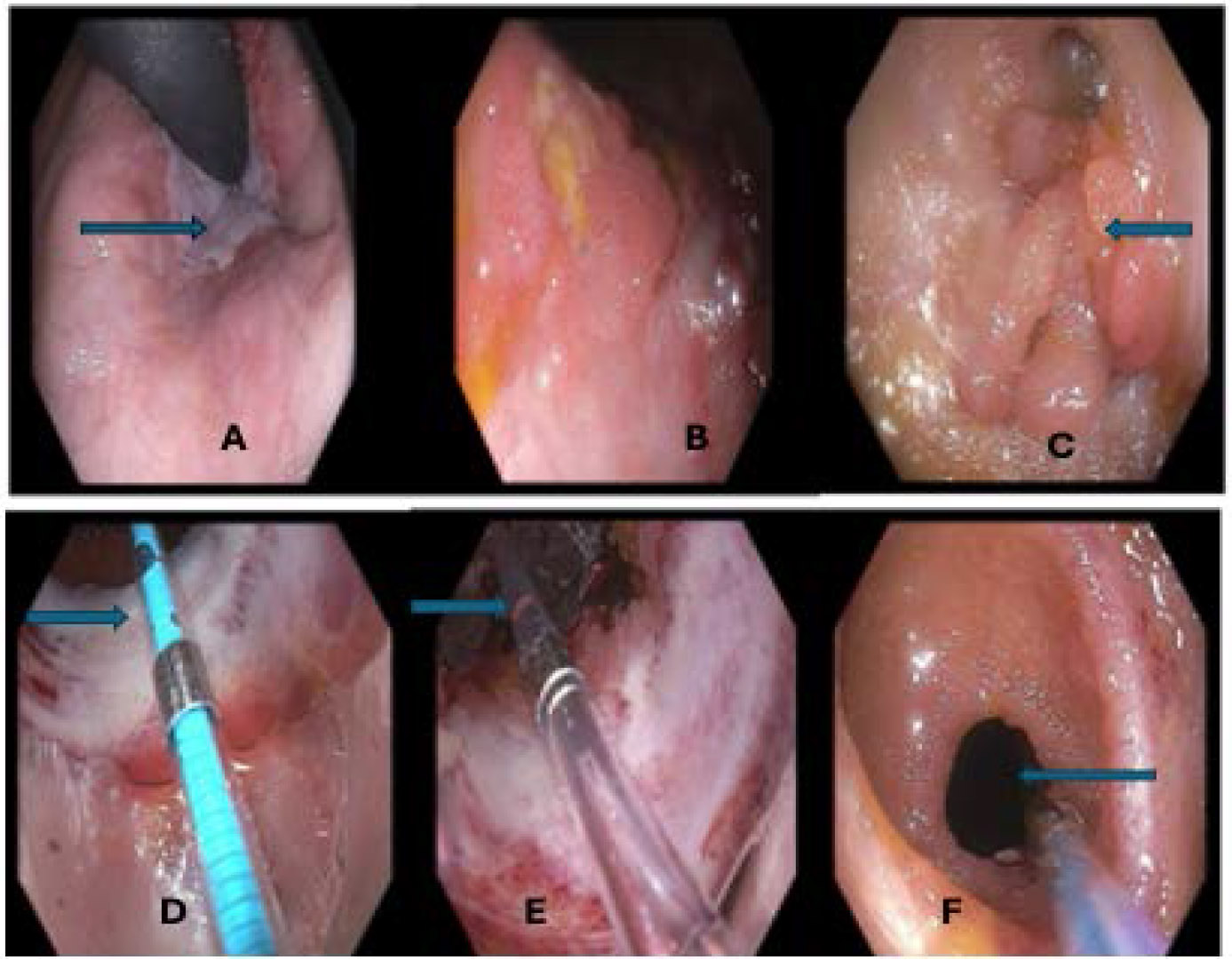
Endoscopic images of terminal ileum, stricture and polyps. The arrow in A indicates the ileum stricture (A, B); the arrow indicates the polyps of the ileum (C); the arrows indicate the dilation of the ileum stricture using the balloon dilator (D, E); the arrow indicates the ileum opening (F).
